# Practical and Pharmaceutic Remarks on the Veratrum Viride

**Published:** 1835

**Authors:** 


IV. *Practical and pharmaceutic remarks on the Veratrum Viride.*—In No. 32 of the Amer. Journal, the profession are favored with a paper by Dr. Osgood, of Rhode Island, on the Veratrum Viride, or "American hellebore, swamp hellebore, Indian poke, Indian uncas, poke-weed, bear-weed, itch-weed, tickle-weed." We have copied out the whole of the catalogue of popular synonims, that our non-botanical brethren (unfortunately by far too numerous) may perchance be directed, by some one of them, to a knowledge of the plant itself. The *Veratrum Viride* appears to be an active medicine. According to professor Tully, as quoted by our author, it is, 1st. deobstruent or alterative, (rather indefinite for the 19th century;) 2d. acrid narcotic; 3d. emetic; 4th. epispastic; 5th. errhine. Passing over a commentary by Dr. Osgood on each of these properties, we shall present our readers with an account of some experiments made by him and one of his friends, on themselves.

"At 12 o'clock, M. I took two grains of the finely pulverized extract. At 1 began to experience a slight sense of uneasiness at the stomach, but not amounting to nausea. This uneasiness at the stomach, though so slight as to be attended with very little inconvenience, continued till about half past 1, when vomiting commenced. The contents of the stomach were thrown off without nausea, but with a sense of rising in the œsophagus, which perhaps might be compared to the rumination of animals. Judging from my sensations at the time, should suppose the muscular fibres of the stomach contracted gradually and steadily upon its contents, until they were expelled, the diaphragm and abdominal muscles remaining entirely inactive. After the vomiting had continued a considerable length of time, it appeared to be more the effect of spasmodic action, and was attended with chills and coldness of the whole body, but moisture of the skin. At the expiration of about an hour, vomiting ceased, and was followed by dimness of sight, dilatation of the pupils, vertigo, faintness and somnolency, pulse at the wrist 40 in the minute, and scarcely perceptible. I then took 25 M laudanum, and fell asleep. After the lapse of an hour, awoke with a continuance of the same symptoms, together with a dull pain in the epigastrium, and immediately repeated the laudanum. But finding no relief, the dimness of sight increasing, and on motion of the body, or turning the head, amounting almost to blindness, a sensation of stiffness in the voluntary muscles supervening, particularly the temporal and extensors of the head, together with considerable general prostration, the dose of laudanum was doubled. This produced a partial abatement of the symptoms, and after another similar interval was repeated, with half a gill of brandy, which soon effected entire relief. In connection with these symptoms, it should be observed that I am unusually susceptible to the operation both of narcotics and emetics.

The individual to whom I have alluded as also taking this extract, may perhaps be considered as at the other extreme in the range of susceptibility. He commenced at 9 o'clock in the evening, with two grains. In ten or fifteen minutes' slight uneasiness at the stomach; at half past 9 took four grains more; at 10, a sensation of something like a ball rising in the œsophagus, which seemed to extend up as far as the top of the sternum, as if propelled by a gradual tonic contraction of the stomach. At quarter past 10 vomiting commenced. This was attended with very little inconvenience at first, but after continuing a short time became more severe, the ejections consisting principally of bile; together with the vomiting, there was much ineffectual retching; almost constant hiccough; chillness; dimness of sight; vertigo; inability to control the voluntary muscles; distress at the stomach; pulse small and creeping, and 34 in a minute; the ordinary frequency ranging from 56 to 58. As these symptoms were becoming more aggravated, he took ʒss. of laudanum, and went to bed scarcely able to walk. In ten or fifteen minutes the laudanum was repeated, which soon produced sleep. In the morning was apparently in better health than he had been for several months. At 7 the same morning, three grains more were taken; at 9 complained of a confused sensation in the head, and almost an entire loss of power of the gastrocnemii muscles. At 12 M. three grains more were taken, and at half past 12, all the muscles of the forearm were affected in the same manner. At 1, vomiting; pulse 40, and other symptoms essentially the same as the day before, excepting a less degree of chilliness. At half past 2, took 45 M laudanum, and in the course of two hours the effects of the medicine entirely subsided, excepting the inability of using the gastrocnemii muscles. At 11 in the evening two grains more were taken, which, in about three quarters of an hour, produced vomiting like the other cases, but without any appreciable narcotic effect." p. 302–3.

The following observations present it, as a valuable medicine in a group of very painful and too often refractory diseases:

"It is unnecessary fully to enter upon a therapeutic application of this article, or to enumerate all the diseases in which it has been employed. Among those in which it stands foremost in the list of remedial agents, are the arthritic inflammations. In this class of diseases, it should be given in such doses as at first fall short of producing disturbance of the stomach, as one-third of a grain of the extract, or ʒss. of the tincture, regularly repeated every three or four hours, and gradually increasing to the extent of producing narcosis or vomiting on the one hand, or resolution of the disease on the other. To insure its best effects, opium in moderate quantities should be used. A combination of the wine, with the tincture of opium, in the proportion of three parts of the former to one of the latter, cannot, it is said, be distinguished in its operation from the celebrated *eau medicinale*, excepting by the catharsis which sometimes ensues from the use of the latter. This combination is much more efficient than the wine or tincture alone, producing less disturbance of the stomach, and can be employed in larger quantities without inconvenience from its narcotic effects. In *gout*, of the regular kind, this article manifests its best powers. It is the opinion of Dr. Tully, that with proper management it will cure a majority of cases. It proves most successful in those constitutions which are not impaired by habits of gluttony and intemperance, at the same time it is much better adapted to broken down constitutions, than the colchicum or veratrum album, on account of the exhaustions these articles are liable to produce. If used in efficient doses, and perseveringly continued several days, there are few cases but will be decidedly benefitted, if not radically cured. It appears to be as well adapted to rheumatism as gout. In the treatment of that disease, both in its acute and chronic form, the article is well worthy the attention of the profession. There is no remedy in the materia medica within my knowledge, with the exception, perhaps, of the actæa racemosa, to which *acute rheumatism* more easily yields. In this disease it should also be combined with opium, for the purpose of relieving pain, and qualifying its effect upon the stomach. The amount of opium conjoined, should be graduated in some measure by the severity of the pain. Thus qualified, it should be administered at regular and short intervals, generally as often as every three hours, in such doses as at first fall short of producing nausea, and gradually increased. Thus administered, the system is kept under its steady and uniform influence. If the doses fall short of producing its specific effects upon the stomach and brain, or if administered so irregularly that the effects of one dose pass off before another is given, but little will be accomplished. In a common case of acute rheumatism, a cathartic of calomel should first be premised, unless the bowels are in a relaxed condition, or some other circumstance exists to contravene this practice. If the stomach is not in an irritable state, it is then best to commence with f. ʒj. of the tincture, in the combination recommended in gout, each dose to be increased 5 or 10 M, as the case may require, till some effect is produced. All local applications should be avoided, as in no way promoting the operation of internal means, and only liable to draw the disease from one part to another, where perhaps its presence is still more to be dreaded. The more acute the disease, the more erratic in its character, and the earlier in its progress, the more speedily does it yield to this course of medication. In *chronic rheumatism*, unattended with inflammation and swelling of the joints, it is less successful than in the acute, from its being a less controllable form of the disease. In this variety, however, it is probably more efficient than any other remedy of equal safety which we possess. It is often necessary to continue its use several days before much benefit is perceived." p. 305–6.

We shall conclude this notice with a paragraph on the pharmacy of our medicine.

"The pharmaceutic preparations of this plant are tincture, wine, extract, ointment, infusion, decoction, and powder of the root. Of these, the tincture, wine, extract, and ointment, are the most eligible forms, and for common medicinal purposes appear to be all that are requisite.

The tincture is prepared by adding the recent bruised root, ℥vj. to diluted alcohol, Oj. I was formerly in the habit of using ℥viij. to the pint, but this appears to be more than is necessary for saturation; medium dose from f. ʒss. to f. ʒj. For the wine, recent bruised root, ℥vj.; white wine, ℥xiv.; officinal alcohol, ℥ij. The alcohol is necessary to prevent the preparation from becoming sour in warm weather; dose the same as of the tincture. In reference to the relative value of these two preparations, I do not know that any thing can be said in favor of the wine, which would not with equal truth apply to the tincture. In medicinal efficacy, there appears to be no appreciable difference. The extract is made simply by expressing the juice of the recent root, and inspissating in the sun. Thus prepared, it is hard and dry, of a grayish color, and capable of being reduced to an impalpable powder. It requires a considerable quantity of the root to produce much of the extract. To obtain the juice it must be strongly bruised and subjected to strong pressure." p. 304.

